# Taste matters: lessons learned in translating a conditioned drug cue reactivity paradigm from rodents to humans

**DOI:** 10.3389/fpsyg.2026.1724256

**Published:** 2026-03-18

**Authors:** Roberto U. Cofresí, Julián A. Aponte Zabala

**Affiliations:** Department of Psychological Sciences, University of Missouri, Columbia, MO, United States

**Keywords:** alcohol, aversive, liking, pavlovian, wanting

## Abstract

**Introduction:**

Bidirectional translation between human and animal models of drug-predictive cue reactivity may accelerate biomarker and treatment development for substance use disorders. We report findings from a pilot study in which *de novo* cue conditioning procedures from non-human animal models of drug (here: alcohol) cue reactivity were adapted for neuroimaging and psychophysiology experiments with human subjects.

**Methods:**

Participants were 10 healthy young adults (60% female, age 21–23 yr) who could undergo electroencephalogram (EEG) recordings and functional magnetic resonance imaging (fMRI) and reported binge drinking at least once in the past 3 months. Prior to the experiment, participants were informed that they would be consuming sips of a strong, sweet mixed drink (10–15% ethanol v/v in 15% w/v sucrose). We hypothesized that prior real-world experience with alcohol, including anticipation of pleasurable post-ingestive psychoactive effects, might shape a person’s hedonic and incentive responses to a new visual cue for an unfamiliar alcohol beverage. Consequently, we expected to observe conditioned liking of the alcohol-paired cue, motivated attention as indexed by the amplitude of the late positive potential (LPP) in cue-locked EEG segments, and reward-network engagement (fMRI). Participants also underwent visual cue pairings with sips of sugar water (15% w/v sucrose).

**Results:**

Most participants showed conditioned disliking of the alcohol-paired cue. While they exhibited more motivated attention to the alcohol-paired than to the unpaired control cue, only the left orbitofrontal cortex was activated more strongly by the alcohol-paired than by the unpaired cue. In contrast, most participants showed conditioned liking of the sugar-paired cue and more motivated attention to it than to its unpaired control cue, as well as broader engagement of the brain’s reward network (e.g., bilateral anterior cingulate, anterior insula, and orbitofrontal cortex).

**Discussion:**

This pilot study demonstrated the feasibility of developing human laboratory experimental paradigms that closely mirror non-human animal models of alcohol and other drug cue conditioning. Yet the pilot study’s unexpected results suggest that for orally administered drugs like alcohol, taste reactivity may matter as much for *de novo* cue conditioning in human subjects as in non-human animal models, even when those human subjects report prior experience with the drug or positive drug expectancies.

## 1 Introduction

Alcohol and other addictive substances are associated with numerous harms to individuals and society at large (Degenhardt and Hall, 2012; Griswold et al., 2018; Rehm et al., 2009; Whiteford et al., 2013). An estimated 400 million adults (≈7% of persons above age 15) worldwide meet the diagnostic criteria for an alcohol or other addictive substance use disorder (SUD) (World Health Organization, 2024). Among the most clinically compelling aspects of SUD are affective–motivational symptoms such as hypo-sensitivity to natural rewards and hyper-reactivity to drug rewards or their learned antecedents (Koob and Volkow, 2010; Volkow et al., 2016). Mutual-aid groups (e.g., 12-step programs) may benefit participants by alleviating these symptoms (Kelly and Greene, 2013), and pharmacological (e.g., naltrexone, buprenorphine) and psychological (e.g., cognitive-behavioral therapy) interventions directly or indirectly target these symptoms (Potenza et al., 2011; Volkow and Blanco, 2023). Yet, all have limited efficacy, and the overarching problem of addiction persists. Prevention- and treatment-relevant discoveries in non-human animal model-based addiction science have failed to yield new solutions (Field and Kersbergen, 2019; Müller, 2018); the same can be said for other neuropsychiatric disorders (McArthur, 2017; McGinty et al., 2024).

One contributing factor to this situation might be that promising discoveries from non-human animal (e.g., rodent) laboratories are rarely verified in human laboratories prior to clinical testing (Nieto et al., 2021; Venniro et al., 2020). This has led to calls for bidirectional translational efforts earlier in the “bench-to-bedside” pipeline (Batman and Miles, 2015; Ray et al., 2021). One area of addiction science that is well-suited for these bidirectional translational efforts is the study of affective–motivational symptoms of SUD, such as hyper-reactivity to drug reward-predictive cues. This is because affective–motivational reactivity to reward-predictive cues is at least partly mediated by brain systems that have been highly conserved across vertebrate evolutionary history, such as the reward network (Haber and Knutson, 2010; Heilbronner et al., 2016; O’Connell and Hofmann, 2011). Indeed, work in non-human animal models suggests that individual differences in Pavlovian conditioned responses to cues and the structure or function of the brain reward network (e.g., pre-existing abnormalities, acquired adaptations) may indicate or contribute to the risk for the development and maintenance of addictive behaviors (Flagel et al., 2009; Flagel and Robinson, 2017; Saunders and Robinson, 2013).

These promising discoveries have yet to be translated and verified in humans (Colaizzi et al., 2020). Nonetheless, translational efforts are ongoing. This is true for natural reward-based (Colaizzi et al., 2023; Heck et al., 2025) and drug-based Pavlovian conditioning (Field and Duka, 2001, 2002; Kareken et al., 2012; Mayo and De Wit, 2015; Mayo and de Wit, 2016; Oberlin et al., 2018).

### 1.1 The current study

Building upon aforementioned translational efforts, in this report, we present results from a neurobehavioral human laboratory pilot study of visuo-gustatory conditioning using alcohol and sugar. Our primary objective is to demonstrate the feasibility of a translational paradigm for ingested drug cue conditioning and share lessons learned from our initial approach. To provide context for these lessons, we outline below the rationale for a critical choice in the paradigm: the use of a novel non-preferred alcohol beverage, and what we expected to observe as a result.

#### 1.1.1 Rationale for choice of alcohol for conditioning and expected results

Through direct (experiential) and indirect (non-experiential) learning, people become aware of the acute effects produced by alcohol ingestion and associate these affectively charged outcomes with antecedent stimuli. Positively valenced acute outcomes are believed to be the ultimate reinforcer for alcohol use, at least initially (George and Koob, 2017; Koob and Volkow, 2010) if not throughout the history of alcohol use (Cho et al., 2019; King et al., 2021, 2022, 2025), and their anticipation is widely considered one of the drivers of alcohol seeking (Cox and Klinger, 1988; Oei and Baldwin, 1994) *via* appetitive motivational processes such as craving and desire (Christiansen et al., 2016, 2017; Johnson and Fromme, 1994; Leeman et al., 2009). These learned outcome expectancies, which can be activated by both discrete and contextual antecedent stimuli (e.g., the sight or smell of a preferred alcohol beverage, typical alcohol self-administration time, places, prior knowledge about alcohol availability, or intentions to consume alcohol at an event), form the basis of placebo manipulations in different types of alcohol use-related laboratory studies involving human subjects. Expectancy-based placebo effects can be so remarkably powerful that people behave as if, and report feeling, intoxicated (McKay and Schare, 1999; Schlauch et al., 2010). Thus, prior knowledge that alcohol will be consumed in a laboratory experiment may be sufficient to override unconditioned responses to a novel (non-preferred) alcohol beverage and shape newly conditioned responses to align with positively valenced expected psychopharmacological effects.

Consequently, we expected that study participants would show conditioned liking of, and motivated attention to, the ethanol–sucrose receipt- compared to omission-predictive visual cue, as well as engagement of the brain’s reward system. We expected similar effects for the appetitive control condition: sucrose receipt- compared to omission-predictive visual cue. In this study, conditioned liking was assessed using self-report (ratings) before and after cue conditioning. Motivated attention to the visual cues was indexed using the magnitude of the P3 or late positive potential (LPP) component of the cue onset event-related potential (ERP) in task-based EEG. The greater the extrinsic (e.g., task-based) or intrinsic (e.g., affective, biological) motivational significance of the eliciting stimulus, the larger the magnitude of this ERP component (Begleiter et al., 1983; Cuthbert et al., 2000; Schindler and Straube, 2020; Schupp et al., 2000; Squires et al., 1977); effects believed to be driven by attentional mechanisms (Becker and Shapiro, 1980; Hajcak et al., 2006, 2013; Schupp et al., 2007). Motivated attention was assessed before, during, and after conditioning. Engagement of the brain’s reward system was assessed before and after cue conditioning using task-based fMRI.

Ethanol–sucrose and sucrose solutions were used as the liquid outcomes for cue conditioning for several reasons. First, to avoid brand, category, or product-specific effects on affective–motivational responses (Boyland et al., 2024, 2025), including those indexed by ERP components (Bosshard et al., 2016; Handy et al., 2010; Thomas et al., 2013). Second, to align with work in rodent labs, many of which continue to use sweetened ethanol solutions or sweetener fading procedures to overcome alcohol refusal (Calleja-Conde et al., 2023; Faccidomo et al., 2025; Hosová and Spear, 2017). Third, to align with placebo-controlled alcohol administration studies in humans, where people are asked to consume a novel (non-preferred) alcohol beverage, typically a sweetened high-concentration ethanol solution (e.g., Corbin et al., 2021; Doty and de Wit, 1995). Fourth, to exert experimental control over orosensory stimulus features and the blood alcohol concentration (BAC) time course across study participants.

## 2 Method

### 2.1 Participants

The planned sample comprised young adult women and men aged 21—25 years who lived within or near a Midwestern college town community in the USA, were able to read and write in English, and reported at least one binge-drinking episode (≥4 alcoholic drinks in 2 h for women; ≥5 for men) in the past 3 months. Since prior work suggests links between alcohol sensitivity and alcohol cue reactivity (Cofresí et al., 2025), efforts were made to include individuals with extreme (high or low) scores on the Alcohol Sensitivity Questionnaire (ASQ) (Fleming et al., 2016). Several exclusion criteria were established for the planned EEG and MRI procedures: uncorrected vision problems; impaired smell or taste; chronic or major neurological or psychiatric illnesses; non-removable ferrous metal or electronic devices in the body or head; hairstyles preventing adequate scalp access for EEG sensors; head circumference too large (>65 cm) or too small (<50 cm) for our EEG sensor nets; or a body mass index suggesting obesity (≥30 kg/m^2^). Due to the planned alcohol administration component, we also excluded individuals who reported current involvement in Substance Use Disorder (SUD) treatment programs, were actively trying to cut down or abstain from alcohol or other addictive drug use, had medical contraindications to alcohol consumption, or experienced strong “flush” reactions. Female participants could not be pregnant or nursing.

Recruitment was accomplished using community-wide digital advertisements (e.g., events newsletter), reposted in multi-week waves, complemented by physical flyers. Participants completed a prescreening survey and videocall with study staff prior to visiting the lab for enrollment. The survey assessed eligibility criteria, sociodemographic information, and alcohol use.

With respect to alcohol use, we assessed binge drinking frequency, likelihood of alcohol use disorder (AUD), sensitivity to acute effects of alcohol, and anticipation of specific acute effects. Binge drinking frequency was probed using a single item (“During the last 3 months, how often did you have 5 or more (for men) or 4 or more (for women) drinks containing any kind of alcohol within a two-hour period?”) with response options ranging from “1 or 2 days” to “every day” (coded as 0.016 to 7 days per week, respectively). The likelihood of AUD was captured by total scores on the Alcohol Use Disorders Identification Test (AUDIT), a validated 10-item self-report instrument (Bohn et al., 1995; Saunders et al., 1993) that queries both the intensity of alcohol use (e.g., “Over the past 12 months, how often did you have six or more drinks on one occasion?”) and use-related problems (e.g., “Over the past 12 months, how often have you failed to do what was normally expected of you because of drinking?”) using standardized response options. Applying contemporary AUD terminology (DSM, 2013) to the graded severity scoring algorithms for the AUDIT (Babor and Robaina, 2016), total AUDIT scores > 7 indicate possible mild AUD, > 15 indicate possible moderate AUD, and > 19 indicate likely severe AUD. Sensitivity to different acute effects of alcohol (e.g., feeling “buzzed” or “relaxed,” experiencing “blackout” or “hangover,” etc.) was captured on the Alcohol Sensitivity Questionnaire (ASQ), a validated 15-item self-report instrument (Fleming et al., 2016). ASQ total scores (higher scores indicate that more drinks are needed to experience any given acute effect from drinking, reflecting lower sensitivity) were obtained by back-transforming z-scores derived from a standardized person mean imputation scoring method used to account for links between drink counts and experienced acute effects (Lee et al., 2015). The scoring algorithm was performed separately for males and females to account for sex-typical differences in alcohol pharmacokinetics (Gandhi et al., 2004). Finally, anticipated specific acute effects of alcohol were probed using the six-item Brief Biphasic Alcohol Effects Scale (BBAES) and two items from the Drug Effects Questionnaire (DEQ), which are validated self-report instruments for capturing anticipated or actual effects for a given dose of alcohol (Fridberg et al., 2017; Morean et al., 2013; Rueger et al., 2009; Rueger and King, 2013). Participants completed the anticipated BBAES first. They were instructed to consider the extent to which they would expect to feel “energized,” “excited,” “sedated,” “slow,” “sluggish,” or “up” shortly after consuming 2 standard alcohol drinks (defined for them as 14 g absolute alcohol equivalents: e.g., 12 fl. oz. of beer, 1.5 fl. oz. of rum, vodka, or whiskey, etc.) and mark their response on Likert scales (0-10; anchors: “not at all” to “extremely”). Next, the two items from the anticipated DEQ (“At that time, I would like the effects that I would be feeling” and “At that time, I would want to consume more standard alcohol drinks”) were presented with a visual analog scale (0–100; anchors: “not at all” to “very much”). Responses to BBAES items “energized,” “excited,” and “up” were summed to form the Stimulation subscale score, and responses to BBAES items “sedated,” “slow,” and “sluggish” were summed to form the Sedation subscale score.

The final sample size for the present report was *N* = 10 (see Table 1 for descriptives). These individuals completed an early version of our laboratory’s classical visuo-gustatory conditioning protocols. Based on our experience with this pilot study, protocol modifications were made and implemented for subsequent work in our laboratory (not reported here). Participants were compensated 60 USD for their time in the lab, in cash, after each visit. Additionally, a 60 USD lump sum bonus for completing the 3-day protocol was given after the third lab visit.

**Table 1:** Sample descriptives.

Characteristics	*n*	*%*	*M*	*SD*	Min	Max
Sex						
Female	6	60%				
Male	4	40%				
Race						
White	10	100%				
Ethnicity						
Hispanic or Latino	1	10%				
Not Hispanic or Latino	9	90%				
Student status						
Undergraduate student	9	90%				
Graduate student	1	10%				
Handedness						
Right-handed	8	80%				
Left-handed	1	10%				
Ambidextrous	1	10%				
Age, yr			22.18	0.86	21.31	23.99
Weight, kg			69.02	13.29	45.35	96.17
Height, m			1.73	0.13	1.57	1.93
Body Mass Index, kg/m^2^			22.85	2.63	17.16	25.97
Alcohol Use Behavior			*M*	*SD*	*Min*	*Max*
Binge Days Per Week			1.47	1.04	0.08	3.50
AUDIT-T			10.90	5.88	5.00	25.00
ASQ-T			5.50	1.48	4.00	9.14
ABBAES						
Stimulation			15.90	7.08	3.00	24.00
Sedation			5.80	7.07	0.00	22.00
ADEQ						
Like it			74.00	20.68	33.00	100.00
Want more			68.70	14.02	49.00	100.00

Binge days per week = in past 3 months, weekly frequency of 4 + standard drinks (14 g absolute alcohol equivalents) in 2 h for females, and 5 + standard drinks in 2 h for males. AUDIT-T = Alcohol Use Disorders Identification Test Total Score. ASQ-T = Alcohol Sensitivity Questionnaire Total Score, back-transformed drink counts from sex-stratified standardized person-mean imputation scoring (Lee et al., 2015). ABBAES = Anticipated Brief Biphasic Alcohol Effects Scale. ADEQ = Anticipated Drug Effects Questionnaire. ABBAES and ADEQ were anchored to “shortly after consuming 2 standard alcohol drinks.”

### 2.2 Procedures

**Figure 1:**
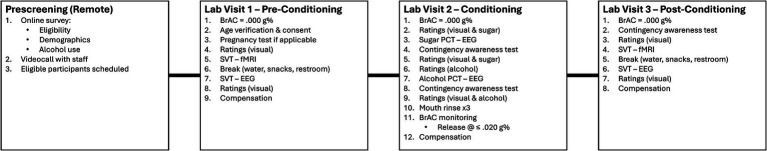
Study timeline. Please refer to the main text for methodological details. Lab visits took place on consecutive days at approximately the same time of day for each participant. BrAC = breath alcohol concentration. The online survey assessed alcohol sensitivity, hazardousness of alcohol use, and anticipated or expected alcohol effects (e.g., stimulation vs. sedation). Ratings = liking (visual analog scale). SVT = shape viewing task [event-related design, passive (no manual response requirements)]. fMRI = functional magnetic resonance imaging. EEG = electroencephalography. Sugar = 15% sucrose w/v in distilled water. PCT = Pavlovian conditioning task (discriminative conditioned visual stimuli, intra-oral unconditioned stimulus delivery). The contingency awareness test = rating (on visual analog scale) of perceived unconditioned stimulus probability following specific visual stimuli. Alcohol = 10–15% ethanol v/v and 15% sucrose w/v in distilled water. Mouth rinse = rinsing the mouth with distilled water to remove residual oral alcohol prior to BrAC monitoring (testing every 15 min). Compensation = cash payment for time in the lab.

Figure 1 provides an overview of study-related procedures. Participants completed three laboratory visits scheduled on consecutive days. Visits took place during weekdays and started between 11 a.m. and 4:30 p.m. Care was taken to schedule lab visits for the same time of day each day to minimize within-person diurnal variability in physiology. Participants were instructed to abstain from alcohol and other drug use (except caffeine and nicotine) for 24 h and to refrain from eating for several hours before lab visits (2 h for visits 1 and 3, 4 h for visit 2). Sobriety was verified at the start of each lab visit by testing for zero (0.000) breath alcohol concentration (BrAC) (Alco-Sensor VxL; Intoximeters Inc., MO, USA).

At lab visit 1, age was verified prior to informed consent by reviewing government-issued identification documents presented by the participant. All participants were asked to void their bladder and provide a urine specimen. Female participants’ urine was tested for pregnancy (Sure-Vue Stat hCG; Thermo Fisher Scientific Inc., MA, USA). Participants then completed the Shape Viewing Task (SVT) while undergoing fMRI and then repeated the task while their EEG was recorded. Participants also rated the stimuli used in the SVT before the MRI and after the EEG. These procedures provided baseline measures of behavioral and neural responses.

Lab visit 2 involved stimulus ratings and completion of the experimental Pavlovian Conditioning Task (PCT). Participants always completed the sugar PCT first and the alcohol PCT second to avoid alcohol intoxication effects on sugar-related learning. Following the alcohol PCT, participants rinsed their mouths vigorously with distilled water three times, and their BrAC was tested. BrAC was tested every ~15 min thereafter until it was <= 0.020, at which point participants were free to leave the lab.

Lab visit 3 involved completing key procedures from lab visit 1 (ratings, fMRI SVT, EEG SVT) a second time to assess changes in behavioral and neural responses from pre- to post-conditioning.

All procedures were conducted at the University of Missouri Cognitive Neuroscience Systems core facility and were approved by the local institutional ethics review board prior to data collection.

#### 2.2.1 Electroencephalography (EEG) procedures

##### 2.2.1.1 Acquisition

EEG was recorded using 256-channel HydroCel Geodesic Sensor Nets (Ag/AgCl electrode pellets, KCl-soaked sponges) connected to a Net Amps 400 amplifier (>1 GΩ input impedance, ≥90 dB common-mode rejection, ≥120 dB isolation-mode rejection; MagStim EGI Inc., OR, USA). The sampling rate was 1 kHz (24-bit A/D, 4 kHz anti-aliasing filter). The online reference electrode was located at Cz, while the isolated common (“ground”) electrode was positioned at a midline location between CPz and Pz. Sensor impedances were kept below 50 kΩ. A chinrest/headrest stabilized the person’s posture and distance from the stimulus presentation screen throughout the recording.

##### 2.2.1.2 Processing

EEG data were preprocessed in Matlab version 2024a (Mathworks Inc., MA, USA) using two open-source packages: eeglab version 2024.1 (Delorme and Makeig, 2004) and erplab version 12.01 (Lopez-Calderon and Luck, 2014). A standard preprocessing pipeline was applied. Data were downsampled to 256 Hz and re-referenced to the average of two mastoid-adjacent electrodes (TP7, TP8). A montage of 64 electrodes at 10–10 locations (Acharya et al., 2016; Nuwer et al., 1998) was extracted for consistency with prior (Fleming et al., 2021) and ongoing EEG conditioning work in the lab. A bandpass filter was applied (0.1–30 Hz, 2nd order Butterworth), DC bias was removed, and line noise (60 and 120 Hz) was attenuated. Artifacts such as blinks, eye movements, and generic discontinuities were identified using independent component analysis (ICA) (Mognon et al., 2011) and removed (*M* ± *SD*: 8.22 ± 3.74 artifactual ICs). Excessively noisy electrodes were removed before ICA and interpolated using the spherical spline method after ICA (*M* ± *SD*: 5.97 ± 2.79 electrodes). EEG segments (−500 to 2000 ms relative to target visual stimulus onset) were extracted and baseline-corrected (−500 to 0 ms). Additional artifact detection routines (e.g., absolute threshold, moving window peak-to-peak, sample-to-sample) were applied to identify and reject segments containing residual artifacts on any electrode. Averaging across participants’ artifact-free segments (PCT *M* ± *SD*: 46.64 ± 2.53 out of 60 total per trial type; SVT *M* ± *SD*: 14.22 ± 2.06 out of 18 total per trial type), the grand average event-related potential (ERP) was derived, and its topography and time course were examined. As expected based on the literature (Hajcak and Foti, 2020), the P3/LPP component (henceforth: LPP) was observed as a positive slow wave unfolding from 300 to 900 ms relative to stimulus onset over occipital, posterior temporal, parietal, and central sites. The time window used for mean amplitude scoring of the LPP component was 400–800 ms to minimize overlap with earlier and later components visible in the waveform. The electrodes over which it was scored were those at which the component was maximal (Cz, CP1, CPz, CP2, P3, P1, Pz, P2, P4, PO7, PO3, POz, PO4, PO8, O1, Oz, O2). For each trial type, mean amplitude scores across the 17-electrode cluster were averaged to maximize measurement reliability (Cofresí et al., 2022b).

#### 2.2.2 Functional magnetic resonance imaging (fMRI) procedures

##### 2.2.2.1 Acquisition

A 3 T Prisma scanner and 32-channel headcoil (Siemens Healthineers AG, DE) with padding to restrict head movements were used to collect images of each participant’s brain. Structural images were acquired using a high-resolution, T1-weighted magnetization-prepared rapid gradient echo (MPRAGE) sequence (TR = 2,300 ms, TE = 2.32 ms, flip angle = 8°, 192 slices, 1-mm isotropic voxels, FOV = 256 mm). To measure task-related blood oxygen level-dependent (BOLD) responses, functional images were acquired using a T2*-weighted, simultaneous multi-slice (SMS) echo-planar imaging (EPI) sequence (multi-band acceleration factor = 3, TR = 2000 ms, TE = 36 ms, flip angle = 70°, 69 slices, 2.2-mm isotropic voxels, FOV = 207 mm).

##### 2.2.2.2 Processing

All images underwent standard preprocessing in Matlab version 2024a (Mathworks Inc., MA, USA) using the statistical parametric mapping (SPM) package version 12 (Penny et al., 2007). Functional images underwent spatial realignment, correction for B0 field map distortion, and slice timing correction prior to spatial co-registration with structural images, segmentation, and normalization to Montreal Neurological Institute (MNI) space using forward deformations with resampling to 1.5-mm^3^ voxels, followed by smoothing with a 6-mm^3^ full-width at half maximum (FWHM) Gaussian filter.

### 2.3 Materials

#### 2.3.1 Visual stimuli (cues)

As in (Cofresí et al., 2022a), visual stimuli consisted of 10 color-filled simple geometric shapes (e.g., cyan hexagon, magenta circle, yellow square). Four shapes were used as conditioned stimuli (CS) in the PCT. One CS (henceforth: CS+) predicted the occurrence of the unconditioned stimulus (US; intra-oral liquid delivery). One CS (henceforth CS-) predicted non-occurrence of the US. Different shape pairs were used for each PCT and counterbalanced across participants. The remaining six shapes were used for the SVT but not PCT, serving as non-conditioned control stimuli (non-CS).

#### 2.3.2 Liquid stimuli (outcomes)

Liquids used as US for the PCT included a standardized sugar solution, 15% sucrose (w/v) in distilled water, and a standardized alcohol solution, 10–15% ethanol (v/v) and 15% sucrose (w/v) in distilled water. Solutions were prepared from commercially available products (e.g., Everclear high-proof grain alcohol [95% v/v ethanol; Luxco Inc., MO, USA], distilled water [Walmart Inc., AR, USA], granulated pure cane sugar [Domino Foods Inc., NY, USA]).

Liquids were delivered intra-orally via an in-house implementation of an open-source, low-cost gustometer design (Canna et al., 2019) consisting of an Arduino Uno Rev3 (Arduino SA, CH), custom code, six peristaltic micropumps (flow rate = 1 mL/s/pump; Gikfun Inc., CN), silicone tubing (2 mm ID x 4 mm OD), and glass reservoir bottles. Plastic connector pieces (3-to-1) linked the inlet tubes from one set of three pumps to one outlet tube and a different set of three pumps to a second outlet tube. Fresh, disposable tube segments served as intra-oral outlet tips for each participant. Plastic in-line check valves were used to prevent backflow. Participants were shown how to place the two outlet tubes bilaterally on the dorsum of the anterior tongue and were asked to hold these in position between their lips during runs of the PCT. To minimize burden, the outlet tubing was supported by semi-flexible modular hose (6 mm OD Loc-Line; Lockwood Products, OR, USA).

#### 2.3.3 Tasks

All tasks were implemented in E-Prime 3.0 (Psychology Software Tools Inc., PA, USA).

##### 2.3.3.1 Shape viewing task (SVT)

A passive picture-viewing paradigm was adapted to obtain pre- and post-conditioning neural measures of visual stimulus information processing. This task consisted of six runs (duration ≈ 3.5 min/run) with intervening rest periods (duration varied by participant but typically ≤1 min). Each run consisted of 30 trials and 30 pre- or inter-trial intervals (EEG version duration = 2–3 s randomly selected in 0.25 s steps within the range; fMRI version duration = 2.5–3.5 s randomly selected in 0.5 s steps within the range), a 2.5 s readiness period before the selection of the first pre-trial interval, and a waiting period following the final trial (EEG version duration = 3 s; fMRI version duration = 6 s). Each trial consisted of a white fixation crosshair (EEG version duration = 0.8–1.2 s trial-wise randomly selected from a uniform distribution; fMRI version duration = 0.5–1.5 s randomly selected in 0.5 s steps within the range) followed by a color-filled geometric shape (fixed duration = 2 s).

Each shape was presented three times per run. Presentation order within runs was randomized across participants and sessions. All stimuli were centrally presented and standardized in size and luminosity. In the EEG recording setup, stimuli subtended 15° horizontal x 9° vertical visual angles, whereas in the MRI scanner, stimuli subtended 4° horizontal x 3° vertical visual angles. To maximize contrast, stimuli were presented on a black background that always filled the screen.

At the pre-conditioning (lab visit 1) SVT, participants were instructed to: “to pay attention to each colored shape and recognize what it represents.” At the post-conditioning (lab visit 3) SVT, participants were additionally instructed to: “keep in mind what you learned yesterday.”

##### 2.3.3.2 Pavlovian conditioning task (PCT)

This task consisted of four runs (duration ≈ 4–5 min/run) with intervening rest periods (duration varied by participant but typically ≤1 min). Each run included 30 trials and 30 pre- or inter-trial intervals (sugar version duration = 3–5 s with random selection from 0.25 s steps in the range; alcohol version duration = 5–7 s with random selection from 0.25 s steps in the range), a 2.5 s readiness period before the selection of the first pre-trial interval, and a 3 s waiting period following the final trial. Each trial consisted of a white fixation crosshair (duration = 0.8–1.2 s, trial-wise random selection from a uniform distribution) followed by a color-filled geometric shape (duration = 2 s).

Each shape was presented fifteen times per run. The visual CS + was followed by intra-oral US nine times per run (60% reinforcement) using a delay conditioning arrangement: US onset at CS offset. US onset latency was ≤25 ms (based on delivery system timing tests). The visual CS- was never followed by intra-oral US. Presentation order within runs was pseudorandomized to avoid consecutive CS + US trials; run order was pseudorandomized across tasks (alcohol vs. sugar); and within- and between-run order was consistent across participants randomized to the same shape-counterbalancing group.

All visual stimuli were centrally presented and standardized in size and luminosity. Stimuli subtended 15° horizontal x 9° vertical visual angles. To maximize contrast, stimuli were presented on a black background that filled the screen.

**Figure 2:**
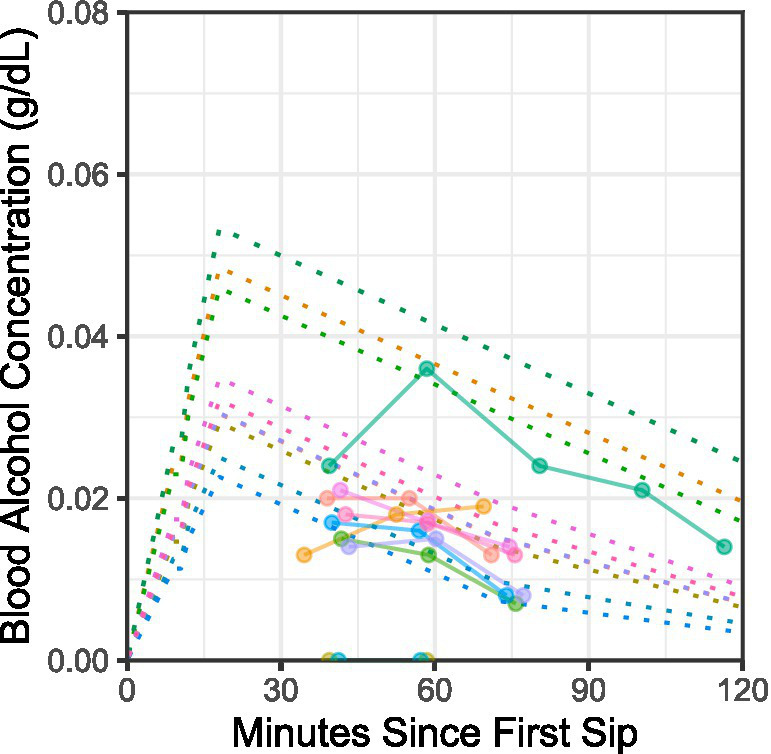
Blood alcohol concentration (BAC) time courses: expected vs. observed. Dotted lines show participant-specific expected BAC time courses estimated using the Matthews and Miller (1979) formula for a hypothetical drinking episode mirroring the amount and timing of alcohol ingestion across the visual cue–intra-oral alcohol sip (10–15% ethanol v/v 15% sucrose w/v solution) pairing procedure in the laboratory. Sip volumes were titrated across participants to target peak expected BAC between 0.020 and 0.060 g/dL. Overlain points connected by lines across assessments show subject-level data (color coding is consistent across panels and figures in the report). Subject-level data are breath alcohol concentration (BrAC) measurements at different times relative to the first sip of alcohol in the laboratory procedure. Participants rinsed their mouths thoroughly three times with water prior to the first BrAC test.

Prior to the task, participants received 1–3 “sips” of the US to train them on how to receive liquid from the intra-oral delivery system. For the sugar US, “sip” volume was 4 mL. For the alcohol US, “sip” volume was individualized for each participant based on their projected BAC time course, given the amount and timing of their alcohol consumption in the task, their biological sex, bodyweight, and the population average alcohol elimination rate (Hustad and Carey, 2005; Matthews and Miller, 1979). In this sample, alcohol “sip” volume ranged from 2 to 8 mL. Expected peak BAC was 0.023–0.053 g/dL at 18 min after the first “sip,” and maximum observed BrAC was 0.010–0.036 g/dL at 34–43 min after the first “sip” (see Figure 2).

At each PCT, participants were instructed: “pay attention to the colored shapes and try to learn which ones, if any, predict liquid delivery into your mouth.” They were also reminded that at any point during a run, liquid flow could be stopped immediately by the experimenter (press of a button) at a participant’s audible or visual request or out of concern for their safety (e.g., due to coughing or choking).

Two out of ten participants requested to stop liquid deliveries during the alcohol PCT: one after the first run and the other after the second run; neither chose to resume liquid deliveries for subsequent runs of the task. Since the pumps controlled by the liquid delivery system could be shut off by the experimenter independently of the computer systems running the PCT and collecting co-registered EEG data, it was possible for these two participants to complete the rest of the alcohol PCT under extinction (non-reinforcement) conditions. Complete behavioral and neurophysiological response data from these two participants are included in the analyses reported herein because they exhibited the modal response pattern despite experiencing fewer reinforced alcohol CS + trials (9–18) than others in the sample (36). These two participants may represent individuals in the population of alcohol users with a higher sensitivity to ethanol’s aversive properties as a tastant.

##### 2.3.3.3 Ratings task

Participants were asked to complete a set of stimulus-related ratings at different points during the lab visits. All stimuli were centrally presented and standardized in size and luminosity. Stimuli subtended 15° horizontal x 9° vertical visual angles. To maximize contrast, stimuli were presented on a black background that always filled the screen, as in the PCT and SVT.

Two sets of items assessed one aspect of participants’ immediate subjective experience of the visual and liquid stimuli: their overt evaluative response to the stimuli. Overt evaluative responses to the visual stimuli were assessed using a univalent item (“How much do you like this stimulus?”) because our prior work indicated that ratings on this item are redundant with ratings on similar items (e.g., “How much do you want this stimulus?,” “How appealing is this stimulus?,” “How pleasing is this stimulus?”) (Cofresí et al., 2022a). One stimulus was presented at a time, along with a visual analog scale (VAS) anchored at 0 (not at all) and 100 (extremely). The order of stimuli presented for rating was randomized. All visual stimuli were evaluated at the start of every lab visit, and at the end of lab visits 1 and 3. The two visual stimuli used for each PCT were also evaluated immediately after the PCT. Immediately before and after each PCT, participants also evaluated the liquid stimulus on the same VAS presented with a similarly phrased univalent item (“How much do you like the liquid stimulus?”).

One set of items assessed participants’ explicit learning: their awareness of cue–outcome contingencies in the PCT. Awareness of cue–outcome contingency was operationalized in simple probability terms: participants estimated how likely each visual cue (CS) was to be followed by a specific liquid outcome (US; alcohol or sugar). One CS was presented at a time, along with a VAS with visual anchors at 0, 25, 50, 75, and 100. The order of CS presented for rating was randomized. Awareness was rated immediately after each PCT administration and the next day.

### 2.4 Analytic approach

#### 2.4.1 Behavioral responses

Contingency awareness was analyzed in two ways. First, two-tailed *t*-tests were used to examine within-subject differences in perceived US probability for the specific US-related CS + vs. CS-. Second, two-tailed *t*-tests were used to examine within-subject differences in perceived US probability for the specific US-related CS + from immediately after conditioning to the next day. US liking was analyzed by using two-tailed *t*-tests to examine within-subject differences in liquid-specific ratings from immediately before to after conditioning. CS liking was analyzed in two ways. First, two-tailed *t*-tests were used to examine within-subject differences in ratings of each cue from before to immediately after conditioning or the next day. Within-subject differences from immediately after conditioning to the next day were also examined. Second, two-tailed *t*-tests were used to examine within-subject differences between US-specific pairs of cues (e.g., alcohol CS + vs. CS-) at specific timepoints. The threshold for statistical significance was *p* < 0.050.

#### 2.4.2 EEG-based neural responses

LPP mean amplitude scores from each SVT EEG recording (lab visit 1, lab visit 3) were analyzed in three ways. First, two-tailed *t*-tests were used to examine the extent to which specific cues (e.g., alcohol CS+) elicited LPP responses that were different from the pre-stimulus baseline within subjects. Second, two-tailed *t*-tests were used to examine within-subject differences between US-specific pairs of cues (e.g., alcohol CS + vs. CS-). Third, two-tailed *t*-tests were used to examine within-subject differences in LPP responses elicited by a specific cue at pre- vs. post-conditioning (lab visit 1 vs. 3, respectively). The first and second analyses were also conducted for LPP mean amplitude scores from each PCT EEG recording (alcohol, sugar; lab visit 2). The threshold for statistical significance was *p* < 0.050.

#### 2.4.3 fMRI-based neural responses

##### 2.4.3.1 First-level analyses

For each SVT fMRI session (lab visit 1, lab visit 3), preprocessed functional images were entered into a first-level general linear model (GLM) to examine the BOLD response to each of ten conditions (i.e., the colored, simple geometric shapes). Each condition was modeled using a delta regressor (duration = 2 s) and convolved with the canonical hemodynamic response function. Intra-run motion was removed using rigid body rotation and translation, and the six resulting motion parameters were included as nuisance covariates. Slow signal drift was attenuated by applying a high-pass filter (128 s, 0.0008 Hz). Contrasts were specified to capture increased activity, on average across runs, in response to alcohol CS + relative to alcohol CS- presentation or alcohol CS- relative to alcohol CS + presentation. Corresponding contrasts were specified involving the sugar CS + and CS-. Additional contrasts were specified to capture increased activity, on average across runs, in response to alcohol CS+, alcohol CS-, sugar CS+, or sugar CS-, relative to the implicit baseline (i.e., activity across fixation crosshair presentations and inter-trial intervals). A contrast similar to the latter was also specified to capture increased activity, on average across runs, in response to non-conditioned shape (non-CS) presentation, by averaging across the six conditions corresponding to the non-conditioned shapes.

##### 2.4.3.2 Second-level analyses

Contrast maps from the first-level GLMs served as input data for second-level GLMs that examined either between-subjects condition differences at a specific session (e.g., alcohol CS+ > CS- at session 2) or within-subjects changes across sessions (e.g., session 2 > session 1 for alcohol CS+). Since the aim of fMRI was to visualize responses in the brain’s reward network, a mask was created using the Wake Forest University PickAtlas tool (Maldjian et al., 2003), with a 2-D dilation factor of 2, and applied to all second-level GLMs. Following an expanded definition of the brain’s reward network (Haber and Knutson, 2010; Huckins et al., 2019; Lesage and Stein, 2016), the mask included the following regions in each hemisphere: amygdala, hippocampus, ventral prefrontal cortex, ventral striatum, and ventral tegmentum–substantia nigra, as well as the anterior cingulate, anterior insula, and dorsal striatum. Due to the preliminary nature of the study and the small sample (*n* = 10), no additional covariates (e.g., age, body mass index, sex, handedness) were included in the second-level GLMs. For each second-level GLM, voxel-wise *p* < 0.001 uncorrected was used to detect significantly activated individual voxels, clusters were defined as spatially contiguous groups of 10 or more individually significant voxels (kE = 10), and cluster-wise *p* < 0.050 uncorrected was used to identify significant clusters. Thresholding parameters were chosen to balance discovery and false positives, given the constraints of a small sample and the exploratory nature of the study. The chosen thresholds can reveal only relatively large “hotspots” of cue-related activation in the brain reward network that are consistent across participants.

#### 2.4.4 Associations with anticipated alcohol effects

Correlation analysis was used to test relationships between self-report measures of anticipated hedonic or incentive responses to real-world alcohol consumption (ADEQ Like It and Want More, ABBAES Stimulation and Sedation) and neurobehavioral measures of hedonic and incentive responses in the laboratory-based alcohol cue conditioning experiment. For efficiency, the latter were person-level difference scores capturing conditioning-related effects, such as differential cue liking (i.e., change in the difference between alcohol CS + and CS- liking ratings from pre- to post-conditioning), differential outcome liking (i.e., change in oral alcohol US liking from pre- to post-conditioning), and differential amplitude of LPP responses to conditioned alcohol cues during conditioning or change from pre- to post-conditioning. Given the small sample, Spearman’s rank correlation coefficients (rho) were used to minimize the impact of overly influential cases or extreme values and to avoid requiring linear rather than merely monotonic relationships between variable pairs. The threshold for statistical significance was *p* < 0.050.

## 3 Results

### 3.1 Cue–outcome contingency awareness

**Figure 3:**
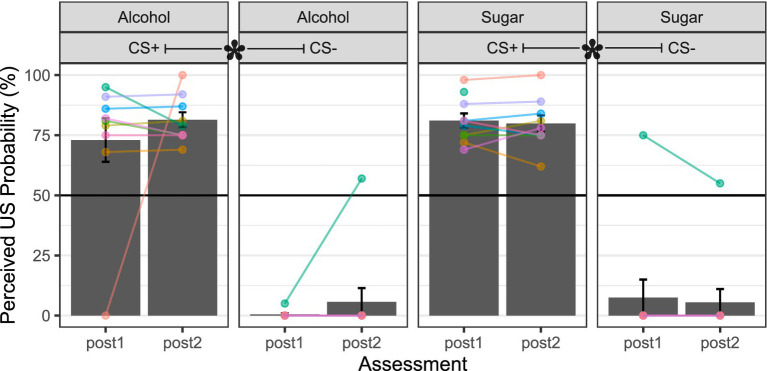
Awareness of cue–outcome contingencies. Alcohol = 10–15% ethanol v/v 15% sucrose w/v solution. Sugar = 15% sucrose w/v solution. US = unconditioned stimulus, the intra-oral liquid outcome (e.g., “Alcohol”). CS + = conditioned stimulus (colored shape) that predicts the occurrence of the US. CS- = conditioned stimulus that predicts the non-occurrence of the US. Post1 = assessment immediately (<5 min) after the cue conditioning (CS–US pairings) episode. Post2 = assessment the next day (24 h). Height of the gray-filled bars in each plot shows sample *M*, and error bars show *SEM*. Overlain points connected by lines across assessments show subject-level data (color coding is consistent across panels and figures in the report). In CS- panels, 9 out of 10 participants rated perceived US probability as approximately 0% at both post1 and post2, so their overlapping subject-level traces appear as a single trace at the floor of each panel. Black-filled asterisk = *p* < 0.05 for within-subject comparison of CS + and CS- at post1 or post2.

Figure 3 shows that, after cue–outcome pairings (i.e., the PCT in lab visit 2), participants knew to expect the alcohol or sugar solution as the outcome following the presentation of the respective CS + and not the CS-, whether assessed immediately after conditioning (*t* ≥ 7.733, *df* = 8–9, *p* ≤ 0.001, *d* ≥ 2.73), or the next day (t ≥ 10.155, df = 8–9, p ≤ 0.001, d ≥ 3.59). Awareness of the intra-oral liquid delivery contingency upon CS + presentation remained unchanged from immediately after conditioning to the next day (*t* ≤ 0.727, *df* = 8, *p* ≥ 0.488, *d* ≥ 0.26). Data from only nine participants were available for tests involving alcohol CS + because one participant consistently skipped the alcohol CS + rating item at both assessments. Data from only nine participants were available for tests involving the sugar CS + at the next-day assessment because a different participant skipping this rating item.

Inspection of subject-level data traces in Figure 3 raised concerns about two persons’ awareness checks: one rated the US probability for alcohol CS + as 0% immediately after conditioning but 100% the next day; the other rated a high US probability for alcohol CS- the day after conditioning and rated a high US probability for sugar CS- at both assessments. The first case is likely due to an attentional lapse or misreading of the rating item. The second case could also be an attentional lapse or generalization from CS + to CS-.

### 3.2 Outcome liking before and after conditioning

**Figure 4:**
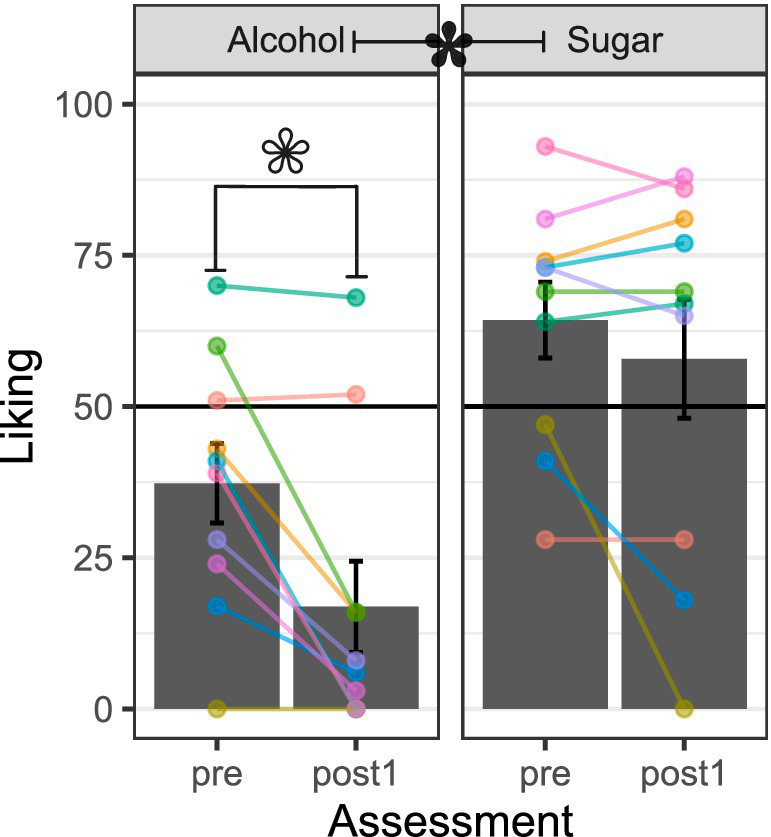
Liking of the intra-oral liquid outcomes. Alcohol = 10–15% ethanol v/v, 15% sucrose w/v solution. Sugar = 15% sucrose w/v solution. Pre = average rating across three assessments: one on the same day as, and immediately before, cue–outcome pairings, as well as two on the day before cue–outcome pairings. Post1 = assessment immediately (<5 min) after cue–outcome pairings. Height of the gray-filled bars in each plot shows sample *M*, and error bars show *SEM*. Overlain points connected by lines across assessments show subject-level data (color coding is consistent across panels and figures in the report). White-filled asterisk = *p* < 0.05 for within-subject comparison of pre to post1. Black-filled asterisk = *p* < 0.05 for within-subject comparison of alcohol US to sugar US at pre or post1.

Figure 4 shows that before the conditioning task, the alcohol solution was moderately disliked (< 50 on the rating scale) and the sugar solution was moderately liked (> 50 on the rating scale). After conditioning, the alcohol solution was liked even less than before conditioning, *t* = −3.74, *df* = 9, *p* = 0.005, *d* = 1.25. Similarly, the sugar solution was liked slightly less after conditioning than before, but the difference was not statistically significant, *t* = −1.20, *df* = 9, *p* = 0.261, *d* = 0.40. Overall, there was a detectable within-subject preference for the sugar solution over the alcohol solution before conditioning, *t* = 3.22, *df* = 9, *p* = 0.010, *d* = 1.07, and afterward, *t* = 3.20, *df* = 9, *p* = 0.011, *d* = 1.07. Inspection of the subject-level data traces indicated that two persons seemed to moderately like the alcohol solution before conditioning and rated it as similarly pleasing after conditioning. Subject-level data traces also indicated that three persons moderately disliked the sugar solution and rated it as similarly or more unpleasant after conditioning.

### 3.3 Cue liking before and after conditioning

**Figure 5:**
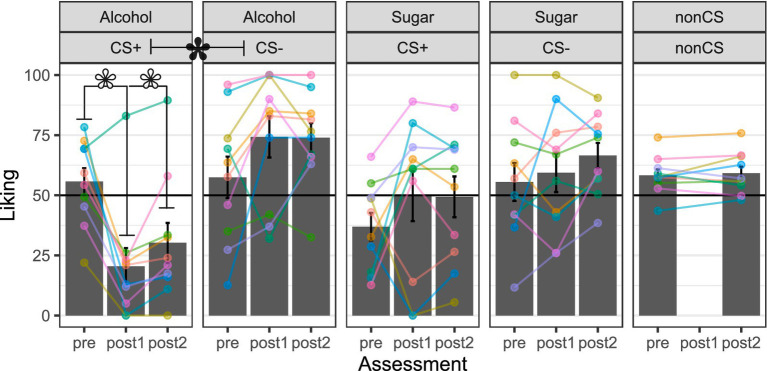
Liking of the visual cues. Alcohol = 10–15% ethanol v/v, 15% sucrose w/v solution. Sugar = 15% sucrose w/v solution. CS + = conditional stimulus (colored shape) that predicts the occurrence of the intra-oral liquid outcome (e.g., “Alcohol”). CS- = conditional stimulus that predicts the non-occurrence of the intra-oral liquid outcome. Non-CS = visual stimuli that did not undergo cue–outcome pairings but served as controls for the effects of repeated exposure (e.g., familiarity, habituation). Pre = average rating across three assessments: one on the same day as, and immediately before, cue–outcome pairings, as well as two on the day before cue–outcome pairings. Post1 = assessment immediately (<5 min) after cue–outcome parings. Post2 = assessment the next day (24 h). Height of the gray-filled bars in each plot shows sample *M*, and error bars show *SEM*. Overlain points connected by lines across assessments show subject-level data (color coding is consistent across panels and figures in the report). White-filled asterisk = *p* < 0.05 for within-subject comparison from pre to post1 or post2. Black-filled asterisk = *p* < 0.05 for within-subject comparisons 0of CS + and CS- at post1 or post2.

Figure 5 shows cue liking ratings before and immediately after conditioning, as well as the next day. The alcohol CS + was liked less, immediately after conditioning than before, *t* = −4.60, *df* = 9, *p* = 0.001, *d* = 1.53. The alcohol CS- was liked more immediately after conditioning than before, although this difference was not statistically significant, *t* = 2.02, *df* = 9, *p* = 0.073, *d* = 0.67. Thus, at the sample level, there was a marked preference for the alcohol CS- relative to CS + immediately after conditioning, *t* = 3.68, *df* = 9, *p* = 0.005, *d* = 1.23. Conditioned preference for the alcohol CS- was still detectable the next day, *t* = 4.24, *df* = 9, *p* = 0.002, *d* = 1.41. Inspection of the subject-level data traces indicated that one person exhibited the opposite pattern.

The sugar CS + and CS- were both liked more immediately after conditioning than before, but the differences were not statistically significant, *t* ≤ 1.30, *df* = 9, *p* ≥ 0.224, *d* ≤ 0.43. The sugar CS + also tended to be liked less than the CS- before conditioning, although this difference was not statistically significant, *t* = −1.86, *df* = 9, *p* = 0.096, *d* = 0.62. Unsurprisingly, no preference for one CS relative to the other was detectable at the sample level immediately after conditioning, *t* = 0.54, *df* = 9, *p* = 0.599, *d* = 0.18, or the next day, *t* = 1.32, *df* = 9, *p* = 0.219, *d* = 0.44. Inspection of the subject-level data traces indicated that immediately after conditioning, three persons liked the sugar CS + less than before, whereas seven liked it more. Subject-level data traces also indicated that immediately after conditioning, three persons liked the sugar CS- more than before, one showed no change, and five liked it less.

Finally, liking ratings remained relatively unchanged across assessments for the non-CS, *t* = 0.64, *df* = 9, *p* = 0.540, *d* = 0.21. Inspection of the subject-level data traces confirmed that roughly half of the persons showed small increases, while others showed small decreases or no change.

### 3.4 Motivated attention to cues during conditioning

**Figure 6:**
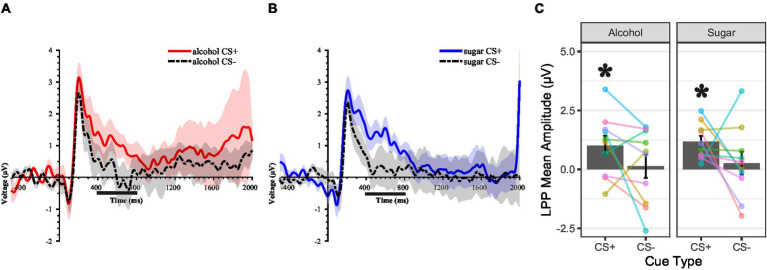
Cue-locked ERPs during cue-outcome pairing procedures showing the cue-locked late positive potential (LPP). **(A,B)** Event-related potential (ERP) waveforms for cue conditions in the alcohol conditioning episode **(A)** and sugar conditioning episode **(B)**, using the averaged signal across a cluster of posterior electrodes over which the LPP was maximal. Lines show the ERP, and colored shading (ribbons) around each line shows the standard measurement error (SME) for that ERP (Luck et al., 2021). A 10-Hz low-pass filter was applied for display purposes only. The time window for LPP mean amplitude scoring is indicated underneath the x-axis. Intra-oral liquid delivery occurred at 2000 ms relative to stimulus onset. **(A–C)** Cue conditions: CS + = conditional stimulus (colored shape) that predicts occurrence of the intra-oral liquid outcome (e.g., “Alcohol”). CS- = conditional stimulus that predicts the non-occurrence of the intra-oral liquid outcome. Alcohol = 10–15% ethanol v/v, 15% sucrose w/v solution. Sugar = 15% sucrose w/v solution. **(C)** Height of the gray-filled bars in each plot shows the sample *M*, and error bars show *SEM*. Overlain points connected by lines across assessments show subject-level data (color coding is consistent across panels and figures in the report). Black-filled asterisk = *p* < 0.05 for within-subject comparison of stimulus-elicited LPP mean amplitude to pre-stimulus baseline mean amplitude.

Figures 6A,B show the CS-locked ERP waveform over posterior electrodes for the alcohol and sugar PCT, respectively. Figure 6C shows the LPP mean amplitude scores. LPP scores were significantly larger for alcohol CS + than the pre-stimulus baseline, *t* = 2.44, *df* = 9, *p* = 0.037, *d* = 1.63, whereas those for alcohol CS- were not, *t* = 0.28, *df* = 9, *p* = 0.784, *d* = 0.19. LPP scores were numerically larger for alcohol CS + than CS-, although the difference was not statistically significant, *t* = 1.60, *df* = 9, *p* = 0.145, *d* = 0.53. Inspection of the subject-level data traces indicated that two persons exhibited the opposite pattern. LPP scores were also significantly larger for sugar CS + than the pre-stimulus baseline, *t* = 4.99, *df* = 9, *p* < 0.001, *d* = 3.32, whereas those for sugar CS- were not, *t* = 0.55, *df* = 9, *p* = 0.595, *d* = 0.37. LPP scores were also numerically larger for sugar CS + than CS-, although the difference was not statistically significant, *t* = 1.51, *df* = 9, *p* = 0.165, *d* = 0.50. Inspection of the subject-level data traces indicated that two persons exhibited the opposite pattern.

### 3.5 Motivated attention to cues before and after conditioning

**Figure 7:**
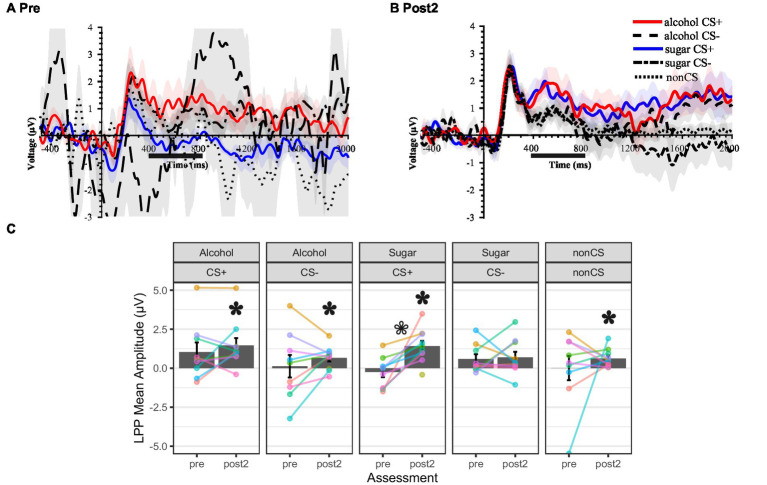
Cue-locked ERPs before and after conditioning showing the cue-locked late positive potential (LPP). **(A, B)** Event-related potential (ERP) waveforms for cue conditions in shape viewing task given the day before cue–outcome pairings (“pre”) **(A)** and the day after cue–outcome pairings (“post2”) **(B)**, using the averaged signal across a cluster of posterior electrodes where the LPP was maximal. Lines show the ERP, and colored shading (ribbons) around each line shows the standard measurement error (SME) for that ERP (Luck et al., 2021). A 10 Hz low-pass filter was applied for display purposes only. Time window for LPP mean amplitude scoring is indicated underneath the *x*-axis. **(A–C)** Cue conditions: CS + = conditional stimulus (colored shape) that predicts the occurrence of the intra-oral liquid outcome (e.g., “Alcohol”). CS- = conditional stimulus that predicts the non-occurrence of the intra-oral liquid outcome. Non-CS = visual stimuli that did not undergo cue–outcome pairings but served as controls for the effects of repeated exposure (e.g., familiarity, habituation). Alcohol = 10–15% ethanol v/v 15% sucrose w/v solution. Sugar = 15% sucrose w/v solution. **(C)** The height of the gray-filled bars in each plot shows sample *M*, and error bars show *SEM*. Overlain points connected by lines across assessments show subject-level data (color coding is consistent across panels and figures in the report). White-filled asterisk = *p* < 0.05 for within-subject comparison of pre to post2. Black-filled asterisk = *p* < 0.05 for within-subject comparison of stimulus-elicited LPP mean amplitude to pre-stimulus baseline mean amplitude.

Figures 7A,B show the stimulus-locked ERP waveform over posterior electrodes for the pre- and post-conditioning SVT, respectively. Figure 7C shows the LPP mean amplitude scores. One participant had too few (<5) artifact-free EEG segments available from the pre-conditioning SVT recording, so they were excluded from analyses involving the pre-conditioning scores.

Before conditioning, none of the cue-related LPP scores differed significantly from the pre-stimulus baseline, *t* ≤ 1.04, *df* = 8, *p* ≥ 0.131, *d* = 1.88, whereas after conditioning, all of them except sugar CS- did, *t* ≥ 2.82, *df* = 9, *p* ≤ 0.020, *d* ≥ 1.88.

Both before and after conditioning, LPP scores were larger for the alcohol CS + than CS-, but in neither case was the difference statistically significant (pre: *t* = 1.89, *df* = 9, *p* = 0.091, *d* = 0.63; post: *t* = 2.16, *df* = 9, *p* = 0.058, *d* = 0.72). Alcohol CS + and CS- LPP scores grew slightly from pre- to post-conditioning, but the difference was not statistically significant (CS+: *t* = 1.10, *df* = 9, *p* = 0.299, *d* = 0.37; CS-: *t* = 1.17, *df* = 9, *p* = 0.271, *d* = 0.39). Inspection of the subject-level data traces indicated that roughly half of the persons exhibited some increase in LPP scores, while others exhibited a decrease or negligible change.

Before conditioning, LPP scores were *smaller* for the sugar CS + than CS-, and this difference was statistically significant, *t* = −2.530, *df* = 9, *p* = 0.032, *d* = 0.84. After conditioning, LPP scores were larger for the sugar CS + than CS-, although the difference was not statistically significant, *t* = 1.52, *df* = 9, *p* = 0.162, *d* = 0.51. There was no apparent change in sugar CS- LPP scores from pre- to post-conditioning, *t* = 0.27, *df* = 9, *p* = 0.790, *d* = 0.09, but there was substantial and statistically significant growth in sugar CS + LPP scores, *t* = 4.05, *df* = 9, *p* = 0.003, *d* = 1.35. Inspection of the subject-level data traces indicated that almost all persons exhibited increases in sugar CS + LPP scores, while seven person exhibited decreases or no change in sugar CS- LPP scores, and three persons exhibited increases in the latter.

Finally, LPP scores also grew slightly across assessments for the non-CS, but the change was not statistically significant, *t* = 0.83, *df* = 9, *p* = 0.427, *d* = 0.28. Inspection of the subject-level data traces indicated that roughly half of the persons exhibited some increase, while others showed a decrease.

### 3.6 Brain reward network regions activated by cues before and after conditioning

Table 2 shows activation clusters detected for outcome-specific cue contrasts at the pre- and post-conditioning fMRI assessments. Before conditioning, no clusters were detected for either alcohol CS+ > CS- activity or alcohol CS- > CS+. After conditioning, the alcohol CS + induced greater activity than CS- at a cluster in the left superior frontal gyrus, orbital part, whereas no clusters were detected where the alcohol CS- induced greater activity than CS + .

**Table 2:** Brain reward network regions differentially activated by conditioned liquid-predictive cues on the day before or after the cue–outcome pairings.

Session	Contrast	Cluster	Cluster size (# voxels)	Activation volume (mm^3^)	MNI Coordinates (X Y Z) for Peak Voxel	Anatomical area of peak voxel
Pre	Alcohol CS+ > CS-	-	-	-	-	-
	Alcohol CS- > CS-	-	-	-	-	-
Post2	Alcohol CS+ > CS-	1	24	81	–14 54 –10	Frontal_Sup_Orb_L
	Alcohol CS- > CS+	-	-	-	-	-
Pre	Sugar CS+ > CS-	1	17	57.375	40 –20 –13	Hippocampus_R
	Sugar CS- > CS+	1	53	178.875	27 12 0	Putamen_R
		2	107	361.125	−38 0 16	Insula_L
		3	19	64.125	38 58 –2	Frontal_Mid_Orb_R
Post2	Sugar CS+ > CS-	1	257	867.375	−46 11 –8 *	Insula_L
					−36 18 –10	Insula_L
					−40 11 –2	Insula_L
		2	58	195.75	36 17 14 *	Insula_R
		3	29	97.875	12 22 28	Cingulum_Ant_R
		4	33	111.375	−16 35 20	Cingulum_Ant_L
					−8 30 23	Cingulum_Ant_L
		5	44	148.5	−20 –7 22	Caudate_L
		6	13	43.875	−26 4 –26	ParaHippocampal_L
		7	46	155.25	−2 29 28	Cingulum_Ant_L
		8	45	151.875	42 22 –12	Frontal_Inf_Orb_R
		9	18	60.75	26 60 –13	Frontal_Mid_Orb_R
		10	75	253.125	46 12 –2	Insula_R
					39 8 –2	Insula_R
		11	12	40.5	−21 56 –8	Frontal_Mid_Orb_L
	Sugar CS- > CS+	-	-	-	-	-

Sessions: “Pre” = the day before cue–outcome pairings and “Post2” = the day after cue–outcome pairings. Contrasts represent session-level averages. No liquid outcomes were presented at either session. CS+ = conditional stimulus (colored shape) that predicts the occurrence of the intra-oral liquid outcome (e.g., “Alcohol”) during cue–outcome pairings on the intervening day. CS- = conditional stimulus that predicts the non-occurrence of the intra-oral liquid outcome during cue–outcome pairings on the intervening day. Alcohol = 10–15% ethanol v/v 15% sucrose w/v solution. Sugar = 15% sucrose w/v solution. MNI = Montreal Neurological Institute. In the anatomical area of peak voxel column, the specific area label taken from the Automated Anatomical Labeling (AAL) atlas (Tzourio-Mazoyer et al., 2002) is presented. Activation volume = cluster size (# voxels) x voxel size (1.5 mm^3^). In cases with multiple peak voxels per cluster, an asterisk (*) in the MNI coordinate column indicates that peak voxel was significantly different from the others at p_FWE_ < 0.050.

Before conditioning, the sugar CS + induced greater activity than the CS- at a cluster in the right hippocampus, whereas after conditioning it induced greater activity at twelve clusters located in the left and right anterior insula, left and right anterior cingulate, left and right middle frontal gyrus, orbital part, left caudate, left parahippocampal area, and right inferior frontal gyrus, orbital part. Before conditioning, the sugar CS- induced greater activity than the CS + at three clusters located in the right putamen, right middle frontal gyrus, orbital part, and left anterior insula. After conditioning, no clusters were detected where the sugar CS- induced greater activity than the CS + .

Table 3 shows clusters that tracked growth in activity induced by specific cues from pre- to post-conditioning. Nine clusters were detected that tracked growth in alcohol CS + induced activity from pre- to post-conditioning, located in the left and right putamen, left and right anterior insula, right anterior cingulate, and right inferior frontal gyrus, orbital part. One cluster was detected that tracked growth in alcohol CS- induced activity from pre- to post-conditioning, spanning the right putamen, anterior insula, and inferior frontal gyrus, triangular part.

**Table 3:** Brain reward network regions showing more cue-induced activation on the day after than before cue-outcome pairings.

Contrast	Cluster	Cluster size (# voxels)	Activation volume (mm^3^)	MNI Coordinates (X Y Z) for Peak Voxel	Anatomical area of peak voxel
Alcohol CS+	1	144	486	27 11 6	Putamen_R
				26 5 –2	Putamen_R
				33 –1 0	Putamen_R
	2	320	1,080	−27 –7 10	Putamen_L
				−27 4 2	Putamen_L
				−30 4 14	Putamen_L
	3	14	47.25	−40 –8 –7	Insula_L/Temporal_Sup_L
	4	441	1488.38	44 10 –4	Insula_R
				36 8 6	Insula_R
				42 20 –7	Insula_R
	5	53	178.875	3 28 28	Cingulum_Ant_R
	6	13	43.875	38 30 6	Insula_R
	7	50	168.75	40 29 0	Frontal_Inf_Orb_R
				34 22 0	Insula_R
	8	26	87.75	−34 11 0	Insula_L
	9	13	43.875	−46 16 –2	Insula_L
Alcohol CS-	1	214	722.25	27 20 6	Putamen_R
				42 22 5	Frontal_Inf_Tri_R
				32 29 4	Insula_R
Sugar CS+	1	47	158.625	−27 2 5	Putamen_L
	2	319	1076.625	−45 11 5 *	Frontal_Inf_Oper_L
				−34 6 10	Insula_L
				−42 17 –8	Frontal_Inf_Orb_L
	3	770	2598.75	30 22 6	Insula_R
				45 10 4	Insula_R
				34 10 8	Insula_R
	4	13	43.875	−20 38 –10	Frontal_Sup_Orb_L
	5	13	43.875	−38 6 −8	Insula_L
	6	17	57.375	45 –10 5	Insula_R
Sugar CS-	-	-	-	-	-
Non-CS	1	17	57.375	−26 5 2	Putamen_L
	2	12	40.5	42 18 8	Frontal_Inf_Oper_R

Sessions: “Pre” = the day before cue–outcome pairings and “Post2” = the day after cue–outcome pairings. Contrasts represent change (post2-pre) in session-level averages for specific cue conditions. No liquid outcomes were presented at either session, but on the intervening day, some cues were paired with outcomes in the lab. CS+ = conditional stimulus (colored shape) that predicts occurrence of the intra-oral liquid outcome (e.g., “Alcohol”) during cue–outcome pairings on the intervening day. CS- = conditional stimulus that predicts the non-occurrence of the intra-oral liquid outcome during cue–outcome pairings on the intervening day. Alcohol = 10–15% ethanol v/v 15% sucrose w/v solution. Sugar = 15% sucrose w/v solution. Non-CS = visual stimuli that did not undergo cue–outcome pairings but served as controls for the effects of repeated exposure (e.g., familiarity, habituation). MNI = Montreal Neurological Institute. In the anatomical area of peak voxel column, the specific area label taken from the Automated Anatomical Labeling (AAL) atlas (Tzourio-Mazoyer et al., 2002) is presented. Activation volume = cluster size (# voxels) x voxel size (1.5 mm^3^). In cases with multiple peak voxels per cluster, an asterisk (*) in the MNI coordinate column indicates that peak voxel was significantly different from the others at p_FWE_ < 0.050.

Six clusters were detected that tracked growth in sugar CS + induced activity from pre- to post-conditioning, located in the left putamen, left superior frontal gyrus (orbital part), left inferior frontal gyrus (orbital part), left inferior frontal gyrus (opercular part), and left and right anterior insula. No clusters were detected that tracked growth in sugar CS- induced activity.

Two clusters were detected that tracked growth in non-CS induced activity from pre- to post-conditioning: one located in the left putamen and the other in the right inferior frontal gyrus (opercular part).

### 3.7 Associations with anticipated alcohol effects

**Figure 8:**
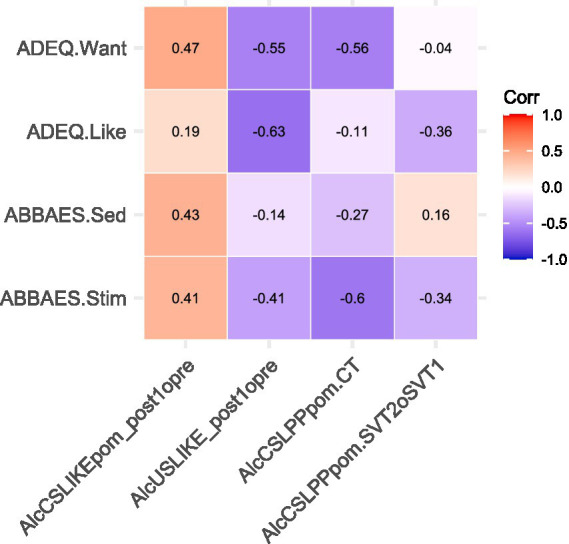
Associations between self-report measures of real-world alcohol effect expectancies and neurobehavioral measures of alcohol cue conditioning. ADEQ.Want = Anticipated Drug Effects Questionnaire (ADEQ) “Want more” item. ADEQ.Like = ADEQ “Like it” item. ABBAES.Sed = Anticipated Brief Biphasic Alcohol Effects Scale (ABBAES) Sedation subscale. ABBAES. Stim = ABBAES Stimulation subscale. AlcCSLike_post1opre = (alcohol CS + liking minus alcohol CS- liking) at post1 minus (alcohol CS + liking minus alcohol CS- liking) at pre. AlcUSLike_post1opre = alcohol US liking at post1 minus alcohol US liking at pre. AlcCSLPPpom.CT = alcohol CS + LPP mean amplitude minus alcohol CS- LPP mean amplitude, both from the alcohol CT. AlcCSLPPpom.SVT2oSVT1 = (alcohol CS + LPP mean amplitude minus alcohol CS- LPP mean amplitude) at SVT2 minus (alcohol CS + LPP mean amplitude minus alcohol CS- LPP mean amplitude) at SVT1. Corr = Spearman’s (rho) rank correlation coefficient. Correlation coefficients are shown inside the cells of the correlation matrix. No correlation coefficients were statistically significant at *p* < 0.05.

Figure 8 shows the correlations between self-report measures of expectancy-like anticipated hedonic and incentive responses to real-world drinking (ADEQ Like, ADEQ Want, ABBAES Stim, and ABBAES Sed) and neurobehavioral measures related to alcohol consumption and cue conditioning in the laboratory. None were statistically significant, yet there was consistency in the direction of association for three of the four laboratory measures examined. Pre- to post-conditioning changes in alcohol CS + relative to CS- liking were positively correlated with all alcohol expectancy-like measures; the largest correlation was with the anticipated positive incentive response to drinking (ADEQ Want: rho = 0.474, *p* = 0.166). In contrast, pre- to post-conditioning changes in alcohol US liking were negatively correlated with all alcohol expectancy-like measures; the largest correlation was with the anticipated positive hedonic evaluation of immediate drinking consequences (ADEQ Like: rho = −0.626, *p* = 0.053). Differential amplitude LPP response to the alcohol CS + relative to CS- during conditioning was also negatively correlated with all alcohol expectancy-like measures; the largest correlation was with the anticipated stimulant-type subjective response to alcohol (ABBAES Stim: rho = −0.595, *p* = 0.069).

## 4 Discussion

To facilitate bidirectional cross-species addiction neuroscience, more human laboratory procedures for modeling relevant behavioral phenotypes, such as affective–motivational reactivity to drug cues, need to mirror rodent laboratory procedures. Here, we report on an initial preliminary study, the primary goal of which was to demonstrate the feasibility of using a visuo-gustatory delay conditioning procedure to alter a person’s affective–motivational response to a visual stimulus by pairing it with the receipt of an ethanol–sucrose or sucrose solution, as typically done in rodent laboratory models of conditioned cue reactivity. Below, we first discuss the results of this study and its limitations. We then consider the lessons it provided, which have informed our current efforts and may aid others in developing human laboratory platforms to test promising discoveries from preclinical non-human animal models of addictive behavior.

### 4.1 Current study findings

The visuo-gustatory delay conditioning procedure was successful, as evidenced by cue–outcome contingency awareness checks (Figure 3), but alcohol-related affective–motivational responses were contrary to our predictions. Most participants liked the intra-oral sucrose outcome but disliked the intra-oral ethanol–sucrose outcome (Figure 4)—a preference pattern that parallels the alcohol vs. sugar taste preferences observed in rodents (e.g., Henniger, 2002; Tordoff, 2002), reinforcing the comparability of humans with our most widely used non-human animal models. After cue–outcome pairings, all but one participant disliked the ethanol–sucrose receipt-predictive visual cue, while most participants liked its omission-predictive counterpart (Figure 5). This conditioned aversion to the ethanol–sucrose receipt-predictive cue was rapid and robust, with two participants displaying it despite choosing to complete the conditioning task in *de facto* extinction after the first 9–18 intra-oral ethanol–sucrose deliveries (others in the sample received the planned 36 intra-oral ethanol–sucrose deliveries across the conditioning task). Conversely, most participants showed increased liking for the sucrose receipt-predictive visual cue and decreased liking for its omission-predictive counterpart at least immediately after, compared to before the cue–outcome pairings (Figure 5). Thus, conditioned evaluative responses to visual cues appeared to follow from the unconditioned evaluative response to the liquid outcome, that is, its affective valence (Christoffersen et al., 2017).

The EEG-derived measure of motivated attention, LPP amplitude, indicated that, as expected, more attention was paid to the liquid outcome receipt-predictive visual cue than to the omission-predictive visual cue during cue–outcome pairings, for both ethanol–sucrose and sucrose (Figure 6C). Enhanced attentional processing of the liquid outcome receipt-predictive visual cues also appeared to be retained to the next day (Figure 7C). These study findings are consistent with prior work showing that affective arousal is more influential than valence in determining the amplitude of the LPP component of the human ERP (Cuthbert et al., 2000; de Cesarei and Codispoti, 2011; Minnix et al., 2013; Rozenkrants and Polich, 2008; Schupp et al., 2000). Additionally, these study findings align with prior human ERP work demonstrating amplitude-enhancing effects of cue–outcome pairings on the cue-elicited P3 or LPP component for both appetitive and aversive outcomes (Bacigalupo and Luck, 2018; Christoffersen et al., 2017; Franken et al., 2011; Viemose et al., 2013). The present study findings are also consistent with ample *in vivo* electrophysiological evidence from non-human animal models regarding cortical neuron responses to conditioned alcohol/drug-predictive cues (e.g., Linsenbardt and Lapish, 2015; Starski et al., 2024; Stringfield et al., 2017) and conditioned reward-predictive cues more broadly (e.g., Petykó et al., 2015; Samuelsen et al., 2012).

The fMRI results suggested that, after conditioning, the sucrose receipt-predictive visual cue elicited greater activity than its omission-predictive counterpart in several regions of the brain’s reward network, such as bilaterally in the anterior cingulate, anterior insula, and orbitofrontal cortex. In contrast, the ethanol–sucrose receipt-predictive visual cue elicited greater activity than its omission-predictive counterpart only in the left orbitofrontal cortex (Table 2). Additionally, within the brain’s reward network, for both the ethanol–sucrose and sucrose receipt-predictive visual cues, there was increased activity in the bilateral anterior insula after compared to before cue–outcome pairings (Table 3). There was also increased activity in the left putamen after compared to before cue–outcome pairings for both liquid outcome receipt-predictive cues; however, this was also true for the control cues that did not undergo cue–outcome pairings (Table 3), suggesting it may be unrelated to cue–outcome memory retrieval. Broadly, these fMRI findings are consistent with prior work demonstrating the involvement of these and other regions of the brain’s reward network in hedonic and incentive processing of both appetitive and aversive stimuli and/or in the preparation of not only approach but also avoidance (defensive) responses (Aupperle et al., 2015; Delgado et al., 2011; Hayes et al., 2014; Metereau and Dreher, 2015; Reynolds and Berridge, 2002).

Lastly, the correlation analyses failed to confirm or disconfirm the hypothesis that anticipation of positively valenced immediate outcomes from alcohol consumption would shape conditioning of positive hedonic and incentive responses to alcohol and thus to the alcohol-predictive cues used in the laboratory (e.g., due to attitude transfer or generalization from positive anticipations or expectancies). Although no correlation test was statistically significant, consistencies in the correlation matrix (Figure 8) suggested that more positive anticipations may have: (i) amplified disliking of alcohol’s taste; (ii) attenuated conditioned disliking of the visual cue for alcohol receipt; and (iii) attenuated motivated attention (LPP amplitude) to the visual cue for alcohol receipt. Patterns (i) and (iii) are contrary to what was predicted based on the hypothesis, while pattern (ii) is only partially consistent. Consequently, the extent to which anticipation of pleasurable post-ingestive alcohol/drug effects may mold hedonic and incentive responses to new alcohol/drug-associated sensory stimuli remains unclear. It may be that stimulus properties (e.g., intensity, dissimilarity) impose constraints. Future studies varying the intensity and familiarity of the sensory stimuli in the conditioning paradigm may be fruitful.

### 4.2 Current study limitations

This study’s findings should be interpreted in light of its limitations. The primary limitation is that it was a preliminary study with a small sample. Small sample sizes are associated with inflated effect sizes (Button et al., 2013) and constraints on generalizability [e.g., due to demographic homogeneity (Table 1)]. A second major limitation is that participants underwent a single, short (20–25 min) episode of visuo-gustatory cue conditioning in the laboratory. In the natural environment of participants’ daily lives, cue–outcome pairings occur across multiple discrete episodes over several days. It remains to be seen if repeated cue conditioning episodes, and consequently, repeated experiences of pleasurable post-ingestive consequences (e.g., “buzzed,” “relaxed”), would alter the affective–motivational response to orosensory or associated visual stimuli. Relatedly, shape–sip pairings in this study were experimentally controlled to produce very low BACs (Figure 2) compared to BACs typically experienced by participants during real-world alcohol consumption. Thus, it remains to be seen whether a controlled delivery of more sips, leading to higher BACs and presumably stronger post-ingestive feelings of subjective stimulation, would prompt at least some participants to change their evaluative responses to the cue when assessed the next day due to naturally occurring post-conditioning revaluation processes (Baeyens et al., 1992; Davey and Mckenna, 1983). A third major limitation is that, although participants were asked to abstain from alcohol and other drug use before study visits, they were not asked to abstain from typical caffeine or nicotine use. Additionally, their breath alcohol was tested to verify sobriety upon arrival, but abstention from other drug use was not biochemically verified. Therefore, effects of caffeine, nicotine, or other drug use at the time of testing cannot be ruled out. Future studies should consider screening for caffeine, nicotine, and other drug use to allow for post-hoc exclusion or sensitivity analyses. A fourth major limitation concerns the ecological validity of the paradigm. Intra-oral liquid delivery was used for three reasons: (i) to minimize movement during EEG recording; (ii) to exert experimental control over orosensory stimulation (e.g., timing relative to visual stimulation, liquid bolus or sip size); and (iii) to employ a strictly Pavlovian procedure (visuo-gustatory stimulus pairings not contingent on subject behavior). However, the intra-oral receipt of liquid stimuli is not natural for humans. Typically, humans use their hands to operate liquid containers and self-administer liquid at a rate of their choosing. The role of agency (choice) and/or embedded skeletomotor response requirements (e.g., use of arm, hand, and fingers to consume the liquid) should thus be examined in future studies, especially as these behavioral elements of the natural human experience of alcohol reward may determine the extent of reward network engagement (Kareken, 2019). Maximizing the ecological validity of the paradigm may be important for enhancing the clinical prognostic or predictive utility of neurobehavioral measures derived from it.

### 4.3 Lessons learned from the current study

#### 4.3.1 Lesson 1: The vehicle for alcohol matters

This preliminary study assumed that by merely informing individuals who regularly engage in risky drinking that they would be drinking alcohol in the laboratory, their affective–motivational reactivity would stem from their tacitly held positive attitudes and beliefs about alcohol and its effects. Specifically, we assumed that participants’ anticipation of positive outcomes from alcohol ingestion in the laboratory would shape their responses to a novel oral alcohol stimulus and its newly learned visual predictor, similar to how alcohol expectancy effects have previously been shown to influence behavioral and physiological responses (McKay and Schare, 1999; Schlauch et al., 2010). However, we found that participants’ responses to the novel oral alcohol stimulus and its newly learned visual predictor were predominantly influenced by their immediate sensory experiences: they disliked this alcohol and its associated visual stimulus (although there was some limited evidence that more positive expectancies may have lessened the conditioned disliking of the associated visual stimulus).

In retrospect, this result is perhaps not surprising. Olfactory and orosensory stimulation with varying concentrations of alcohol is typically evaluated as aversive by humans, likely due to its bitter taste and burning sensations (Nolden and Hayes, 2015). The unpleasant taste of alcohol may explain why many people prefer to consume it in preparations or vehicles that contain additional aromas and flavors (e.g., beer, wine, or mixed drinks), although these may not fully mask the unappetizing taste of alcohol. Similarly, non-human animal laboratories interested in modeling human alcohol use behaviors (i.e., oral self-administration) have long struggled against the unpleasant taste of alcohol, which is perceived even at concentrations as low as 3% v/v by rodents (Kiefer et al., 1990, 1998; Kiefer and Dopp, 1989). To encourage their subjects to overcome taste aversion and discover the rewarding post-ingestive psychoactive effects of alcohol, researchers often add sweeteners (e.g., saccharin, sucrose) and then gradually fade these out (Calleja-Conde et al., 2023; Samson, 1986). The challenges faced in non-human animal laboratories reflect complexities in the human experience. Some people may drink alcohol even if they dislike the taste. Indeed, individuals may differ in their willingness to consume an unpalatable alcoholic beverage when seeking a desired affective outcome, and this individual difference may index risk for acute or chronic problems related to alcohol use. Others may initially dislike the taste of an alcoholic beverage but come to enjoy it later in their relationship with that beverage. These potential between-person differences highlight the conceptual dissociation of liking and wanting at the core of the incentive sensitization theory of addiction (Robinson and Berridge, 1993, 2025) and provide empirical evidence for this dissociation concerning alcohol in humans (Hobbs et al., 2005; Ostafin et al., 2010). The procedures described in this report can be used to obtain behavioral and psychophysiological measures related to these between-person differences. Rodent laboratories already have procedures in place for modeling similar phenotypes, such as aversion-resistant seeking/drinking (De Oliveira Sergio et al., 2023) and conditioned preference/avoidance (Cunningham and Niehus, 1997; Kiefer et al., 1994; Knight et al., 2016; Przybysz et al., 2024). This creates opportunities for bidirectional, cross-species translational research on taste reactivity and its clinical significance (Johansen et al., 2025). Nonetheless, a word of caution is warranted. Intense disgust reactions to the taste of an unfamiliar or non-preferred alcoholic beverage may occur (as presumably happened here for two participants), and this may contribute to study attrition, especially if handled differently than we did (i.e., implementing study procedures in a way that participants could experience fewer reinforced alcohol CS + trials than planned in the protocol while still contributing complete usable data). While intense disgust reactions may reflect naturally occurring individual differences in taste sensitivity among persons who use alcohol, it is a foreseeable risk that should be disclosed to study participants during the informed consent process.

Besides experimenter-prepared ethanol–sucrose solutions and similar options, familiar or preferred alcoholic beverage products can also serve as the intra-oral stimulus for these *de novo* cue conditioning procedures. While using familiar or preferred alcoholic beverages may introduce logistical challenges related to purchasing (e.g., varying costs and availability of different products over time, retailers refusing to accept tax exemptions for research-related purchases) and inventory management (e.g., access control, refrigeration, expiry dates), these challenges are surmountable. From an experimentalist’s perspective, the scientific challenge is that alcoholic beverage products vary in alcohol concentration by volume and across different orosensory dimensions (e.g., smell, taste, texture). This variability can complicate efforts to equate BAC time courses for experiments examining the role of BAC in shaping alcohol-related learning and memory phenomena. Additionally, this variability requires consideration of potential beverage product- or category-specific effects on observed alcohol-related learning and memory phenomena.

To retain control over the features of a familiar oral alcohol stimulus, studies could be conducted using a single beverage product category; however, this approach may be inefficient and have limited generalizability beyond the demographics targeted by manufacturers’ advertising campaigns. Additionally, the orosensory and olfactory stimuli produced by any alcoholic beverage (whether a commercially available beer or the ethanol–sucrose solution used in our study) may not be experienced uniformly across persons, leading to some uncontrolled variance related to person-specific sensory perception (and hedonic evaluation) (Thibodeau and Pickering, 2019). Another option is to assess beverage preferences during screening and stratify the sample to include similar numbers of participants in each beverage category group, with the number of groups determined by generalizability goals and practical considerations. It should be noted that, without an intentional stratification plan, the beverage industry’s marketing efforts may bias the number of represented beverage product categories in a sample based on the demographics of the latter (Cook et al., 2024). Further control over the oral alcohol stimulus features can be achieved by not providing each participant with their specific preferred beverage product, but rather by identifying an exemplar from each beverage product category and providing that exemplar to all participants who indicate a preference for a product in that category. In this case, attention to industry marketing and consumer perceptions is warranted (e.g., light vs. IPA-style beers, red vs. white wine, sweet vs. dry wine), even if branding elements are to be eliminated from the exemplar. However, retaining branding elements may be an important feature in some studies.

#### 4.3.2 Lesson 2: Experimental control in human and non-human animal laboratories

Unexpected results aside, this first study taught our team that it *is* feasible to translate rodent laboratory procedures into human laboratory procedures. Similar to the rodent laboratory, there was experimental control over the frequency and duration of exposure to visual and gustatory stimuli in the human laboratory, and all participants were exposed to the same set of stimuli. As in the rodent laboratory, behavioral and neural responses to the visual stimuli were measured at multiple times before and after pairings with the gustatory stimuli, including the next day, which is critical for assessing the extent to which learning was consolidated into long-term memory, and in contexts where no visuo-gustatory pairings ever occurred (here: the MRI scanner), which is critical for examining the extent to which memory retrieval or expression is context dependent.

Unlike in the rodent laboratory, there was no control over lived experiences outside the laboratory, such as what participants did, ate, or drank before or after their visits. Although we were participant-centered in scheduling laboratory visits (notwithstanding constraints related to scanner and staff availability), participants were not provided housing or transportation to or from the research location. Yet, they arrived on time and completed the three laboratory visits across three consecutive days (100% retention). They also generally adhered to pre-visit procedures, with 97% reporting they had refrained from alcohol use for 24 h and from eating for several hours before lab visits, as well as reporting that they slept their typical number of hours (within-person coefficient of variation across visit days = 10%; between-person *M* ± *SD* = 8.3 ± 0.5 h slept), suggesting they arrived for their visits in comparable physiological states. Although we would like to attribute the high compliance and retention rate to participants’ expressed curiosity about the research and enthusiasm for their role in it, we must acknowledge that they were compensated for their time, in cash, at the end of each laboratory visit. Additionally, we must note that they were healthy emerging adult students, which likely facilitated high compliance and retention.

Finally, unlike work with rodent models, studying human subjects in the laboratory typically requires providing subjects with explicit instructions about study procedures (e.g., “your job is to keep track of the number of times the purple hexagon appears,” “please sit quietly and keep your eyes focused on the fixation cross”). Instructions create opportunities for experimental control over human behavior, including cognition, in the laboratory. In the present study, as part of the conditioning procedures, we instructed participants to pay attention to the visual stimuli and try to learn which ones predict liquid delivery. Although rodents do not receive explicit instructions in conditioning paradigms, they are often presumed to be motivated to explore and engage with their environment, similar to humans. Thus, in some situations, learning antecedents for meaningful events in the environment (e.g., reward availability) may involve goal-driven or purposeful behavior. Our chosen instructions for the conditioning task aimed to provide study participants with a comparable goal and to motivate similar engagement with the task. Were these instructions necessary for the learning observed in the present study? We cannot confirm or disconfirm this possibility because we did not conduct a comparison study using nominal instructions (e.g., “please view the stimuli and consume any liquid delivered into your mouth”) or decoy instructions (e.g., “your job is to count how many visual and gustatory stimuli are presented“). Both explicit (intentional) and implicit (incidental) learning occur in humans, and both may be relevant to various forms of conditioning observed in them (Jones and McLaren, 2009; Moran et al., 2023; Popov and Dames, 2023; Schmidt and De Houwer, 2019). Future studies utilizing this novel alcohol/drug cue conditioning paradigm for the human laboratory should vary instructions to determine the extent to which conditioned responding stems from explicit (intentional) vs. implicit (incidental) learning processes, which might engage different neural mechanisms (Do Carmo-Blanco and Allen, 2019; Köster et al., 2017). Additionally, future studies could consider embedding concealed opportunities for incidental learning into an intentional learning version of the paradigm to gain insight into the possibility that explicit and implicit learning processes operate in parallel during drug use experiences in the natural environment.

### 4.4 Concluding remarks

Despite demonstrating the feasibility of translating rodent laboratory alcohol cue conditioning procedures (e.g., Carpio et al., 2022; Cofresí et al., 2018; Remedios et al., 2014; Tomie et al., 2006) into the human laboratory, the present pilot study suggests that, depending on study goals, it may be necessary to use a familiar or participant-preferred alcohol beverage rather than an unfamiliar one. We assumed that prior life experience with alcohol and accrued positive expectancies would shape binge drinkers’ hedonic and incentive responses to an unfamiliar alcohol beverage in the laboratory. Instead, we found that responses reflected immediate sensory pleasure or displeasure rather than anticipated pleasurable post-ingestive outcomes. These findings suggest boundary conditions on the generalization or transfer of affective–motivational reactivity from alcohol-related stimuli encountered in daily life to alcohol-related stimuli presented in the laboratory setting. Nonetheless, the findings from this pilot study should be considered tentative until replicated in a larger independent sample.

## Data Availability

The raw data supporting the conclusions of this article will be made available by the authors, without undue reservation.
